# Transcriptional Repression of E-Cadherin by Human Papillomavirus Type 16 E6

**DOI:** 10.1371/journal.pone.0048954

**Published:** 2012-11-26

**Authors:** Zarina J. D'Costa, Carol Jolly, Elliot J. Androphy, Andrew Mercer, Charles M. Matthews, Merilyn H. Hibma

**Affiliations:** 1 Virus Research Unit, Department of Microbiology and Immunology, University of Otago, Dunedin, New Zealand; 2 Department of Dermatology, Indiana University School of Medicine, Indianapolis, Indiana, United States of America; University of Birmingham, United Kingdom

## Abstract

There is increasing evidence supporting DNA virus regulation of the cell adhesion and tumour suppressor protein, E-cadherin. We previously reported that loss of E-cadherin in human papillomavirus (HPV) type 16-infected epidermis is contributed to by the major viral proto-oncogene E6 and is associated with reduced Langerhans cells density, potentially regulating the immune response. The focus of this study is determining how the HPV16 E6 protein mediates E-cadherin repression. We found that the E-cadherin promoter is repressed in cells expressing E6, resulting in fewer E-cadherin transcripts. On exploring the mechanism for this, repression by increased histone deacetylase activity or by increased binding of trans-repressors to the E-cadherin promoter Epal element was discounted. In contrast, DNA methyltransferase (DNMT) activity was increased in E6 expressing cells. Upon inhibiting DNMT activity using 5-Aza-2′-deoxycytidine, E-cadherin transcription was restored in the presence of HPV16 E6. The E-cadherin promoter was not directly methylated, however a mutational analysis showed general promoter repression and reduced binding of the transactivators Sp1 and AML1 and the repressor Slug. Expression of E7 with E6 resulted in a further reduction in surface E-cadherin levels. This is the first report of HPV16 E6-mediated transcriptional repression of this adhesion molecule and tumour suppressor protein.

## Introduction

Cervical cancer is the second most common cancer among women worldwide, with over 500,000 new cases being diagnosed annually [1]. The majority of cases of cervical cancer are a consequence of infection with high-risk, oncogenic human papillomaviruses (HPV). These are small, non-lytic, non-enveloped, dsDNA viruses that are tropic for squamous epidermis [2]. The two viral proteins E6 and E7 from high-risk HPV types are the major oncogenes and are necessary for the induction and maintenance of the transformed phenotype [3].

The E6 open reading frame (ORF) encodes an 18 kDa protein containing four Cys-X-X-Cys motifs, which form two zinc finger structures [4]. E6 manipulates a range of cellular functions important in viral genome amplification, replication and persistence in the host, including inhibition of apoptosis as a result of degradation of p53 [5] and increased genomic instability mediated by activation of hTERT [6]. There is increasing evidence that E6 also affects cell adhesion and polarity, via targets such as hDlg, MAGI, hScrib and E-cadherin [7].

E-cadherin, a 120 kDa Type I classical cadherin, is expressed primarily on epithelial cells [8]. It is found on the surface of keratinocytes [9] and Langerhans cells (LC) and E-cadherin-mediated adhesion between these cell types is required for LC retention in the epidermis (49). It is also an important tumour suppressor protein: its loss or inactivation is associated with epithelial-to-mesenchymal transition (EMT), a process involving dedifferentiation, infiltration and metastasis of tumours [10]. Carcinomas of the cervix, as well as cancers from many other tissue types, frequently have decreased or aberrant expression of E-cadherin [11]–[13].

Significantly, it has been shown that E-cadherin expression in the epidermis is reduced or lost during HPV16 infection, which is associated with LC loss at the site of infection [14], [15]. Furthermore, in *in vitro* studies, surface E-cadherin expression is reduced on cells expressing E6 or E7, implicating these proteins in its regulation [14], [16]. Although E7 is reported to repress E-cadherin by augmenting DNA methyltransferase 1 (DNMT1) activity [17], no pathway for E6 regulation of E-cadherin has yet been described. Our objective is to elucidate the mechanism by which E6 regulates E-cadherin, in order to gain an understanding of how HPV16 controls this important cell adhesion and tumour suppressor protein.

## Results

### HPV16 E6 decreases surface and total protein levels of E-cadherin in HCT116 cells

**Figure 1:**
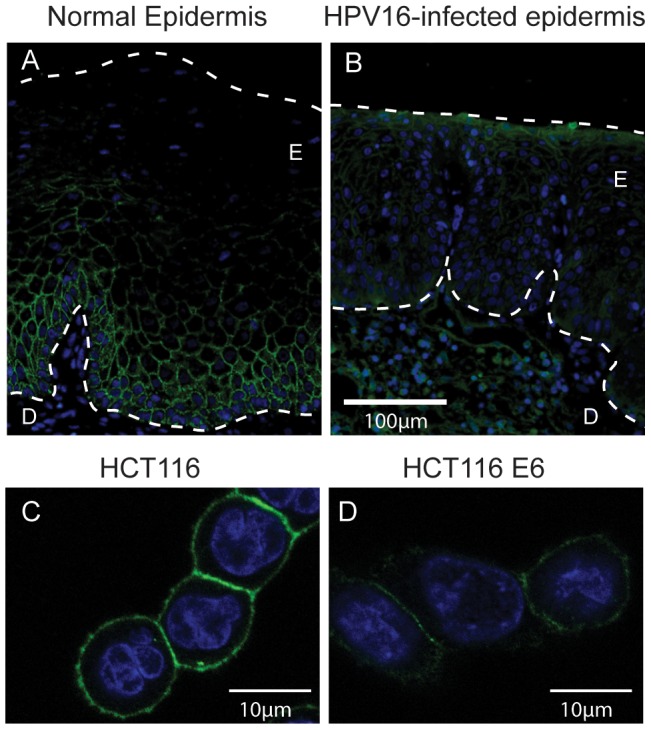
HPV16-infected epidermis displays lowered and irregular E-cadherin staining HPV16 E6 expressing cells have reduced E-cadherin expression. E-cadherin (green) and DAPI stained nuclei (blue) in (**a**) normal and (**b**) HPV16-infected human epidermis. E = epidermis, D = dermis. (**c**) HCT116 and (**d**) HCT116 E6 cells grown on glass coverslips, permeabilised and stained for E-cadherin (green) and DAPI to identify nuclei (blue).

**Figure 2:**
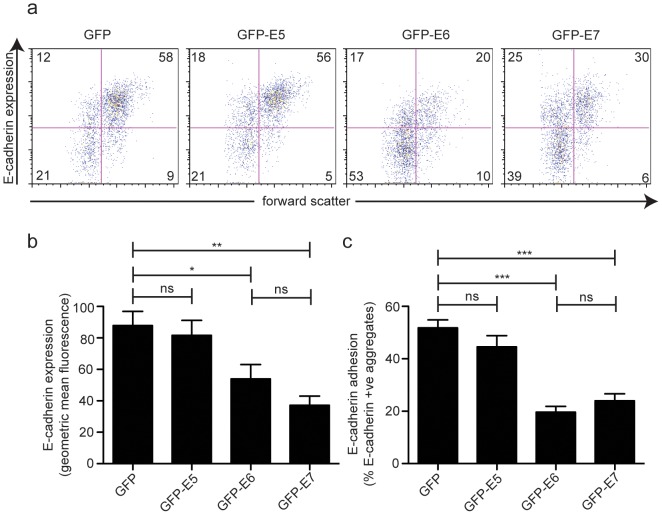
E-cadherin expression and E-cadherin mediated adhesion is reduced in cells expressing HPV16 E6 and E7. HCT116 cells were transfected with plasmids encoding GFP-E5, E6 or E7 and were trypsinised, cleaving surface E-cadherin, 48 h later. Single cell suspensions were cultured for 2 h in low adhesion plates for E-cadherin re-expression and cell-to-cell adhesion to occur. Cells were stained for E-cadherin and GFP positive cells with comparable levels of expression between samples were analysed using flow cytometry. (**a**) Dot plots showing the E-cadherin negative single cell population (*lower left*), E-cadherin positive single cells (*upper left*), E-cadherin positive aggregates (*upper right*), with the percentage of events in each quadrant shown. (**b**) Cell surface E-cadherin fluorescence and (**c**) cellular aggregates mediated by E-cadherin on GFP-positive cells. n = 5 independent experiments. Error bars represent ± SEM. * *P*<0.05, ** *P*<0.01, *** *P*<0.001.

E-cadherin is expressed on the surface of keratinocytes of the basal and suprabasal cervical epidermis (Fig. 1A). In HPV16 infected epidermis, surface E-cadherin expression is lost from these cells (Fig. 1B). We have previously shown that HPV16 E6 expression (transiently) in an immortalized keratinocyte cell line, HaCaT, reduces surface E-cadherin expression by around half [14] and that surface E-cadherin expression is similarly reduced in HCT116 cells stably expressing E6 [18]. HCT116 cells are widely used to study E-cadherin regulation [19]–[21] being intact in the major E-cadherin repressor pathways such as E-box-mediated repression [20] and having low levels of promoter methylation [22]. For those reasons, we chose HCT116 cells for this study. Using immunofluorescence staining and confocal analysis of the HCT116 and E6 cells, visually there was a marked reduction in surface E-cadherin on the cells expressing E6 (Fig. 1c & d). GFP+ HCT116 cells with comparable levels of GFP expression were analysed by flow cytometry for surface E-cadherin following transient expression of GFP, GFP-E5, GFP-E6 or GFP-E7. Transient expression of E6 in these cells similarly reduced E-cadherin by around 50%, comparable to HaCaT cells and to HCT116 cells stably expressing E6 (Fig. 2). E7 was somewhat more effective than E6 in repressing E-cadherin, although not significantly so, and repression of E-cadherin-mediated aggregation was comparable. Consistent with our previous observations in HaCaT cells, E5 had no effect on E-cadherin expression or E-cadherin mediated adhesion.

### The E-cadherin promoter activity is less active and mRNA levels are reduced in E6 expressing cells

**Figure 3:**
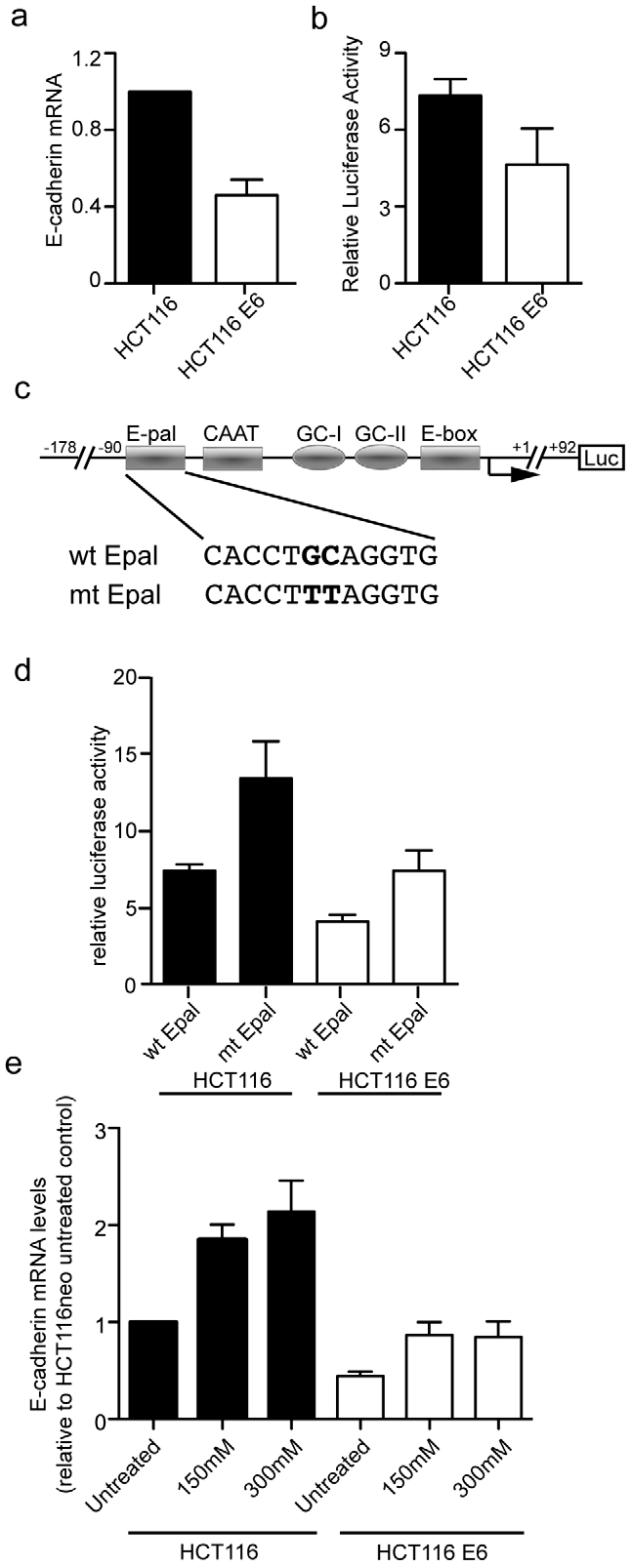
HPV16 E6 reduces E-cadherin protein expression by reducing E-cadherin promoter and transcript levels, independent of the major Epal repressor element and HDAC. (**a**) Levels of E-cadherin mRNA were determined by qRT-PCR analysis on RNA extracted from HCT116 control and HCT116 E6 cells. GAPDH mRNA was amplified in the same RNA samples for normalization. n = 5 independent experiments. (**b**) Luciferase activity of the −178/+92 E-cadherin promoter was measured in HCT116 and HCT116 E6 cells. n = 3 independent experiments. (**c**) Schematic representation of the luciferase plasmid under the control of the E-cadherin promoter. Negative regulatory elements Epal, E-box and positive elements CAAT, GC-I and GC-II are shown as is the mt Epal, with the two-nucleotide inactivating mutation. (**d**) Activity of wild type and mt Epal E-cadherin promoters in HCT116 and HCT116 E6 cells. Luciferase activity of co-transfected pRL-SV40 was used as a control for transfection efficiency and for normalization. n = 8 independent experiments. (**e**) RNA was extracted from HCT116 and HCT116 E6 cells that had been either untreated or treated with HDAC inhibitor, TSA, for 24 h. E-cadherin mRNA levels were measured by qRT-PCR and normalized to GAPDH mRNA levels. n = 3 independent experiments.

Transcriptional repression of E-cadherin has been widely reported in cancer cells, through repression of the E-cadherin promoter, and this might also be the case in cells expressing HPV16 E6. On measuring the levels of E-cadherin transcript in E6 expressing cells we found them to be reduced by around half compared with the parental HCT116 cell line (Fig. 3a). When promoter activity was measured using a luciferase reporter, activity also was reduced in E6 expressing cells (Fig. 3b).

The Epal is a negative regulatory element of the E-cadherin promoter [23], which is bound by several zinc-finger repressors including Snail, Slug and Twist [24]. To determine if the Epal element is involved in E6 repression the E-cadherin promoter, we tested a mutant E-cadherin promoter (mt Epal) [25] that is inactivated by a two-nucleotide mutation in the Epal (Fig. 3c). HCT116 and HCT116 E6 cells were transfected with the wild type or mt Epal E-cadherin reporter plasmids and analysed for luciferase activity. Consistent with the inability of trans-repressors such as Snail, Slug and Twist to bind the mutant promoter, the mt Epal was more active than the wild type E-cadherin promoter in HCT116 cells (Fig. 3b). In cells expressing E6, an increase in activity of the mutant promoter was also observed, however activity was not restored to the level measured in cells lacking E6. From this we concluded that the reduction in E-cadherin transcripts in these cells is not a result of increased repressor activity through the Epal.

### E6 repression of E-cadherin is independent of histone deacetylase activity

Transcription of E-cadherin can be repressed as a result of modifications of histones that cause the chromatin to condense, reducing binding of transactivators and resulting in transcriptional repression. Regulating modifications include methylation or deacetylation, as a result of increased histone deacetylase (HDAC) activity. Histone deacetylation is an important determinant of E-cadherin transcriptional activity [26] and we hypothesized that E6 increases histone deacetylation, thereby repressing E-cadherin transcription. To determine if this was the case cells were exposed to Trichostatin (TSA), an HDAC inhibitor, and levels of E-cadherin transcripts were measured. E-cadherin transcript levels were increased in HCT116 cells treated with TSA, clearly indicating a role for HDAC in repression of *CDH1* (Fig. 3e). However the amount of E-cadherin transcripts following HDAC inhibition increased proportionally in E6 expressing cells. The lack of specific restoration of E-cadherin transcription in E6 treated cells indicates that deacetylation of histones is not significant in the transcriptional regulation of E-cadherin by E6.

### DNMT activity is increased in E6 expressing cells

**Figure 4:**
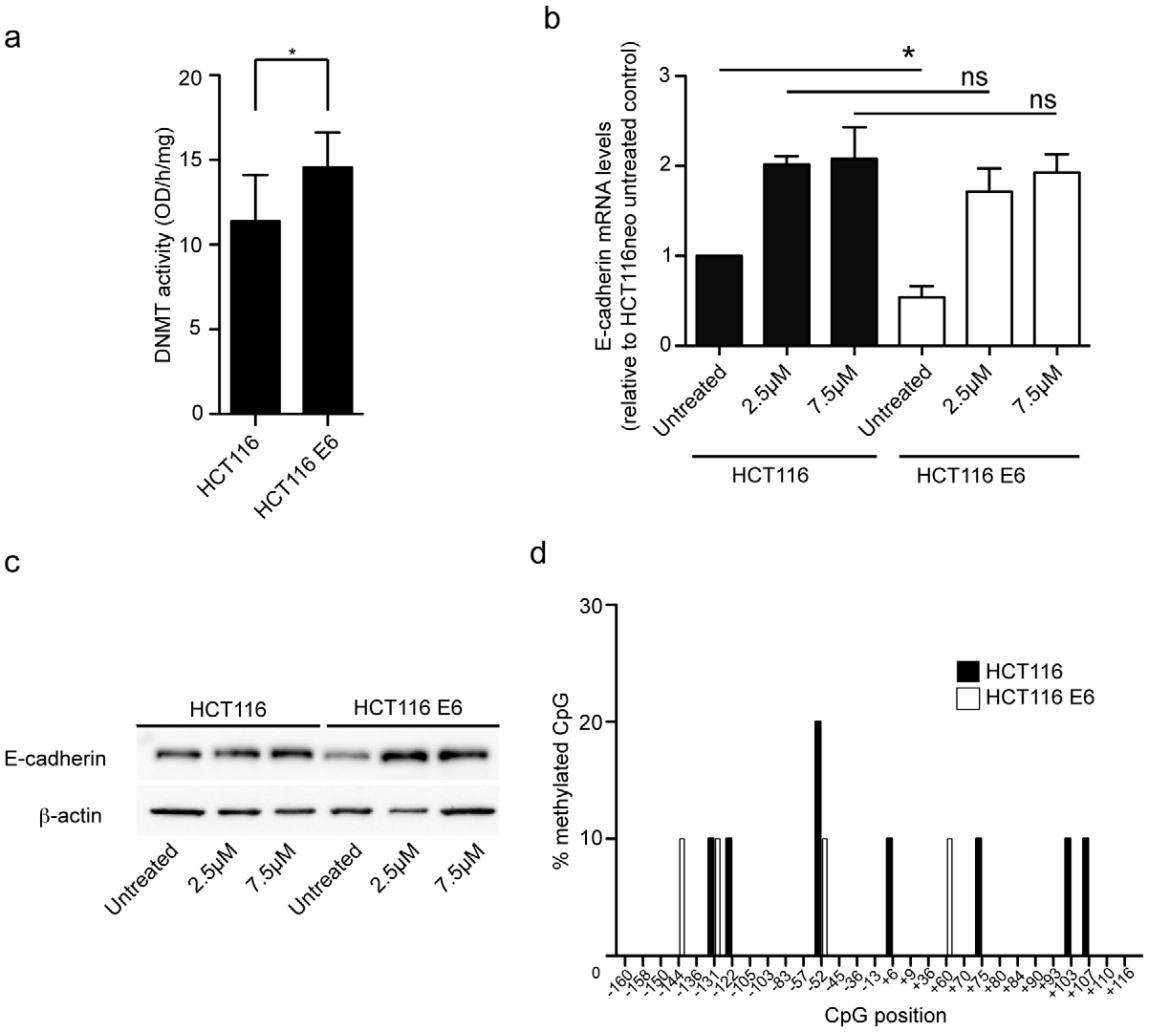
DNMT activity is increased in E6 expressing cells and E-cadherin can be restored following treatment with the DNMT inhibitor, 5-Aza-dC. (**a**) Nuclear extracts from HCT116 and HCT116 E6 cells were assayed for DNMT activity. n = 3 independent experiments. Error bars represent ± SEM. * *P*<0.05. (**b**) RNA was harvested from HCT116 and HCT116 E6 cells treated with 2.5 µM and 7.5 µM 5-Aza-dC for 72 h and analysed by qRT-PCR for E-cadherin mRNA levels, normalized to GAPDH. n = 3 independent experiments. Error bars represent ± SEM. * *P*<0.05; ns = not significant. (**c**) Lysates from HCT116 and HCT116 E6 cells treated with 5-Aza-dC, as above, were tested for E-cadherin expression by western blot. Data representative of three experiments. (**d**) Bisulfite-converted DNA from HCT116 and HCT116 E6 cells was prepared and sequenced to determine the methylation state of CpGs within the minimal promoter region (−191 to +94 bp relative to transcription start site). Nucleotide sequences of ten individual clones of bisulfite converted DNA from each cell line were analysed.

DNMT-mediated hypermethylation of promoter regions can cause transcriptional repression. Increased DNMT activity contributes to a reduction in E-cadherin expression in cells expressing HPV16 E7 and may also be involved in its regulation by HPV16 E6. To test this directly, DNMT activity was measured in nuclear extracts from HCT116 control and E6 cells. We found that activity was increased by around 20% in E6 expressing cells, compared to the control cells (Fig. 4a). Given that this increase, although significant, was modest we examined the effect of 5-Aza-2′-deoxycytidine (5-Aza-dC) inhibition of DNMT on E-cadherin transcription. 5-Aza-dC irreversibly covalently binds to and interferes with DNMT to reactivate hypermethylation-silenced genes [27]. In HCT116 cells E-cadherin mRNA levels were doubled following treatment with 5-AzadC, consistent with some DNMT-specific repression of E-cadherin transcription occurring in these cells (Fig. 4b). In HCT116 cells expressing E6 E-cadherin transcript levels were increased around fourfold, restoring them to the level of treated control cells. This restoration also translated into restoration of total E-cadherin protein levels in E6 expressing cells (Fig. 4c), further supporting the requirement for DNMT in E6 regulation of E-cadherin.

The restoration of E-cadherin transcription to control levels following inhibition of DNMT using 5-Aza-dC supports a methylation dependent mechanism for the regulation of E-cadherin by E6. In order to establish if the inhibitory effect was a result of the E-cadherin promoter itself being methylated in E6 expressing cells, genomic DNA from HCT116 and HCT116 E6 cells was isolated and subjected to bisulfite conversion and sequencing. Consistent with reports by others [22], the parental HCT116 cells showed little evidence of methylation of CpG in the E-cadherin promoter (Fig. 4d). Perhaps somewhat unexpectedly the level of methylation of the CpGs in HCT116 E6 cells did not differ from HCT116 control cells, showing that E-cadherin transcription regulation by E6 is independent of direct methylation of the E-cadherin promoter. From this we conclude that E-cadherin repression is dependent on DNMT through a pathway that is independent of direct methylation of the E-cadherin promoter and that inhibition of DNMT using 5-Aza-dC restores E-cadherin expression.

### The activity of the CDH1 promoter is reduced in E6 cells, which correlates with a generalized reduction in the binding of transcriptional factors to CDH1 in the presence of E6

**Figure 5:**
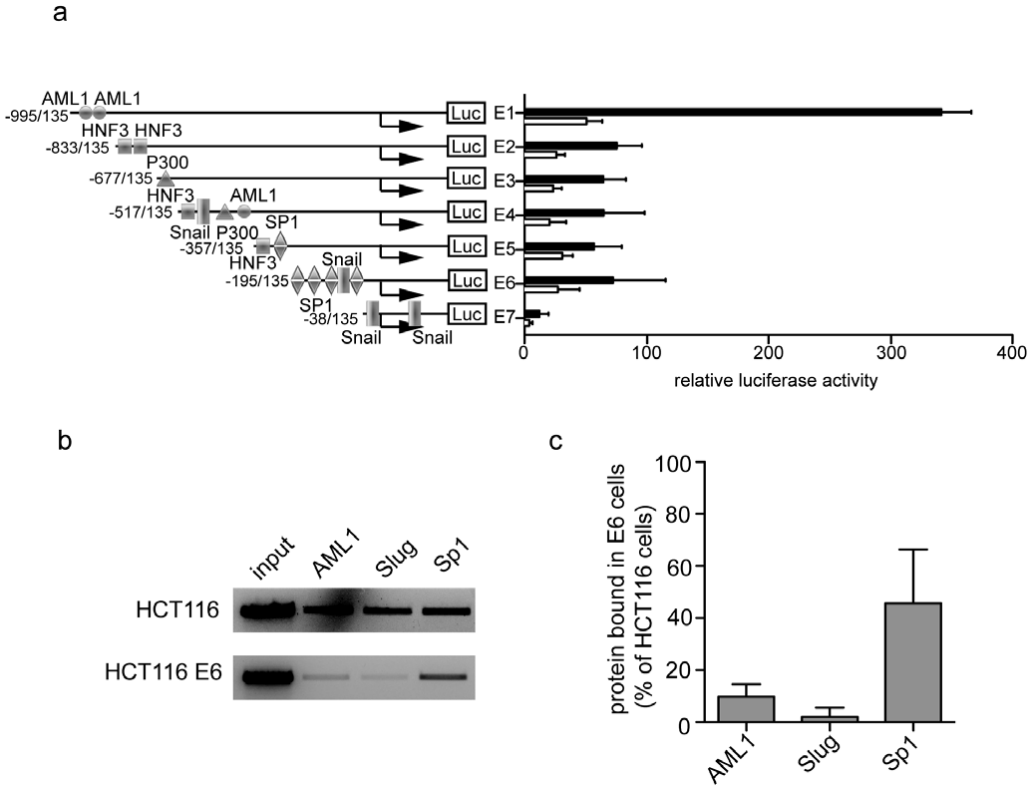
E6 reduces the luciferase activities of the full length and a series of deletion constructs of the human E-cadherin regulatory region, whilst reducing the occupancy of the transcriptional regulators within this region. (**a**) The pGL3 *Firefly* luciferase constructs were transfected into HCT116 (black bars) and HCT116 E6 (white bars) cells and 48 h later luciferase activities were measured. Activity was normalized to pRL-SV40 *Renilla* luciferase activity. Results are from three independent experiments. (**b**) HCT116 and HCT116 E6 cells were subjected to ChIP analysis followed by PCR to determine occupancy of transcriptional regulators AML1, Sp1 and Slug within the E-cadherin regulatory region. (**c**) Three independent experiments were quantified (Image J, NIH) to measure the relative percentage of AML1, Slug or Sp1 bound to the E-cadherin promoter in E6 cells relative to control cells.

We tested an alternative hypothesis, that the increased DNMT activity in E6 cells results in repression of a *CDH1* transactivator, which in turn negatively regulates transcription of E-cadherin in these cells. The E-cadherin regulatory region has several binding sites for activators such as AML1, p300, Sp1 and HNF3 [28]. Repression of E-cadherin transcription by E6 might be a consequence of reduced binding of any of these transcription factors to the E-cadherin promoter. To determine if this was the case, a series of deletion mutants of the 1.2 kb regulatory region of the promoter [28] were tested in order to identify transcriptional activator binding sites that might have a role in its repression. In both control and E6-expressing HCT116 cells the minimal promoter containing only the Epal (Slug and Snail binding) repressor sites (−38/+135) had negligible activity (Fig. 5a). Addition of a further Snail and an Sp1 binding site (−195 to −38) increased the promoter activity in both HCT116 and E6 cells, with the promoter having around a third of the control cell activity in the cells expressing E6. This same trend was observed with no notable change in promoter activity with the addition of a further Sp1 and HNF3 (−357 to −195), Snail (−517 to −357), p300 (−677 to −517) and HNF-3 (−833 to −677) binding elements. However with the addition of the activator AML1 binding sites from position −995 to position −833 there was around a fivefold increase in promoter activity in control HCT116 cells and doubling of promoter activity in HCT116 cells expressing E6. From these data we concluded that the promoter was less active overall in E6 expressing cells and that Sp1 and AML1 binding elements were particularly affected.

We wished to directly determine if the binding of AML1 and Sp1 transcriptional factors to the E-cadherin promoter was reduced. In addition, we chose to measure binding of the Epal-binding Slug repressor to the E-cadherin to consolidate the evidence for the absence of activity through the Epal element. The binding of these three factors to the *CDH1* promoter was analyzed using ChIP. There was around tenfold reduction in the amount of E-cadherin promoter pulled down from E6 cells when an antibody to AML1 was used and around 50% less signal when Sp1 was used in the ChIP assay, compared to the control cells (Fig. 5b & c). In addition to the reduced binding that we observed with the activators, we found that there was negligible binding of the Slug repressor to the E-cadherin promoter in E6 cells whereas Slug binding was detectable in control cells. From this we conclude that the binding of both activators and the repressor protein to the E-cadherin promoter was reduced in E6 expressing cells. These data support reduced access of transacting proteins to the E-cadherin promoter in cells expressing E6, limiting transcription and consequently lowered E-cadherin mRNA levels.

### Co-expression of E7 in E6 cells further reduces surface E-cadherin

**Figure 6:**
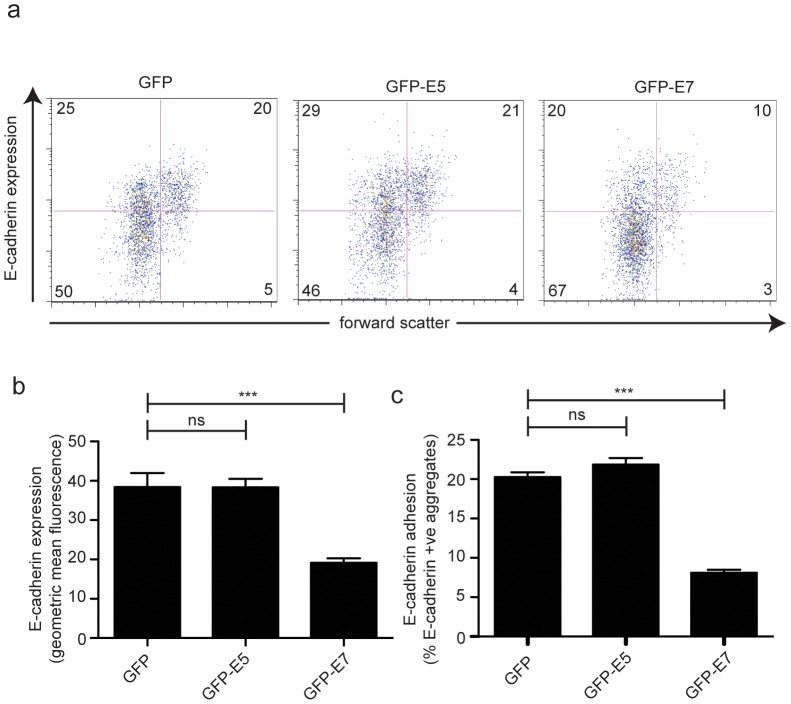
Co-expression of E7 in E6-expressing cells further decreases surface E-cadherin levels and function. HCT116 E6 cells were transfected with GFP, GFP-E5, or GFP-E7 and an adhesion assay followed by flow cytometric analysis of the GFP positive cells was carried out. (**a**) Dot plots showing E-cadherin negative single cell population (*lower left*), E-cadherin positive single cells (*upper left*) and E-cadherin positive aggregates (*upper right*). The percentage of events in each quadrant is shown. (**b**) Cell surface E-cadherin and (**c**) E-cadherin-mediated cellular aggregates were measured. n = 5 independent experiments. *** *P*<0.001.

Given that E7 is also reported to regulate surface E-cadherin, we wanted to determine the effect of its expression on surface E-cadherin in HCT116 E6 cells. HPV16 E7 was transfected in HCT116 E6 cells and the effects on surface E-cadherin were compared with cells transfected with E5 or a control plasmid. As we would predict, expression of E5 had no effect on the amount of E-cadherin on the surface of E6-expressing cells (Fig. 6). In contrast, E-cadherin was further reduced by around half in E6 cells co-expressing E7 and this drop was accompanied by a similar drop in E-cadherin mediated adhesion (Fig. 6b & c). From this experiment we conclude that co-expression of E6 and E7 further reduces levels of cell surface E-cadherin.

## Discussion

In addition to its tumour suppressor function, E-cadherin is an important immune regulatory protein in the epidermis, where it regulates adhesion between LC and keratinocytes. We have previously reported that E-cadherin expression is reduced or lost in HPV infected tissues and that E6 has a role in its reduced expression. In this study we provide the first evidence for reduced levels of transcripts in E6 cells resulting from E-cadherin promoter repression and go some way towards establishing how this regulation might occur.

Key observations in elucidation of the mechanism of E6 regulation of E-cadherin transcription are that DNMT activity is increased in E6 expressing cells and that exposure to an inhibitor of DNMT, namely 5-Aza-dC, restores E-cadherin transcription and levels of the protein. Au Yeung *et al.* (2010) [29] demonstrated a drop of around 30% in DNMT1 protein in CaSki cells and around 60% in SiHa following E6 siRNA knockdown, providing evidence of a role for E6 in increased DNMT1 levels in those cells. Furthermore, Laurson et al. (2010) [17] showed an increase in E-cadherin transcripts following 5-Aza-dC treatment in immortalized keratinocytes expressing HPV16 E6. A primary function of DNMT is to catalyze the transfer of a methyl group to cytosine residues, forming 5-methylcytosines at CpG islands [30] and denying access of transcription factors to their binding sites [31]. Although this would be a plausible explanation for E6 repression of E-cadherin transcription, there was no evidence that the E-cadherin promoter itself was directly methylated in E6 expressing cells. Similarly, an absence of promoter methylation is reported for E7 transcriptional regulation of E-cadherin [17] and consistent with these *in vitro* data, E-cadherin promoter methylation does not appear to occur in low-grade HPV-infected cervical tissue [32]. Overall, there is no evidence to support an increase in promoter methylation mediated by E6 as the cause of the reduced E-cadherin expression observed in HPV16-infected epidermis.

DNMT-dependent general repression of the E-cadherin promoter in E6 may result from increased histone methylation, which can occur independent of DNA promoter methylation [33]. Histones assemble into nucleosomes and are important in epigenetic silencing. Chemical modifications of the amino-terminal tail of histones, such as acetylation or methylation, can affect the access of transcriptional regulatory factors and complexes to chromatin thereby impacting on gene expression [34]. For example, acetylation of the lysine-9 residue on histone H3 leads to the formation of open, transcriptionally active euchromatin whereas methylation at the same residue is associated with inactive heterochromatin [34]. E-cadherin expression is repressed by increased methylation of histones by other proteins, such as enhancer of zeste homolog 2 (EZH2) and Methyl-H3K9-binding protein MPP8 [35], [36]. Increased HDAC activity, leading to inactivation of heterochromatin by deacetylation, did not occur in E6 expressing cells. Our data instead support a methylation dependent modification of histones as a potential mechanism for the repression of E-cadherin transcription. That 5-Aza-dC treatment of cells rapidly reduces lysine-9 methylation at silenced loci, which in turn re-activates silenced genes [34], is in accordance with the reactivation of E-cadherin transcription that we observe following 5-Aza-dC treatment of E6 expressing cells. Further studies are required to provide direct evidence for a role for histone methylation in the repression of E-cadherin transcription in E6 expressing cells.

Binding of several transcription factors was reduced on the E-cadherin promoter in the presence of E6 and there was an associated reduction in promoter activity. Liu *et al* [28] identified binding sites for several transactivators including HNF3, Sp1, P300 and AML1 distal to the transcription start site in the 1.2 kb regulatory region of *CDH1*. In HCT116 cells we observed a marked activation of the promoter on addition of the tandem AML1 sites between −995 and −833, which was not mirrored in cells expressing E6. Also the addition of the Sp1 site proximal to the transcription start activated transcription more effectively in control than in E6 expressing cells. In both cases, binding of the transcription factor to the promoter region was also reduced in cells with E6. Our initial response to these data was that a methylation dependent repression of the promoter of the transcription factor itself might occur, however when we quantified levels of AML1 transcripts in cells with and without E6 there was no difference in levels (data not shown). Similar observations to ours for E-cadherin have been reported for p21^Waf1/Cip1^, where treatment of cells with 5-Aza-dC increased p21^Waf1/Cip1^ in the absence of direct promoter methylation and with no change in histone acetylation [37], [38]. In that case the authors speculated that an increase in responsiveness of the Sp1 activating element of the p21^Waf1/Cip1^ promoter occurred [39]. The effects of 5-Aza-dC treatment on the binding and activity of Sp1 or AML1 to the E-cadherin promoter in E6 cells are yet to be tested but we predict that their binding would be restored by treatment.

The major repressor element of the E-cadherin promoter, the Epal, is bound by a number of repressor proteins including Snail, Slug [40], ZEB1/, SIP1 [41], E12/E47 [42] and Twist [43]. There was no specific restorative effect on E-cadherin promoter activity following mutation of the Epal in E6 cells. We also found that the amount of Slug bound to the E-cadherin promoter was much reduced in E6 expressing cells. This may be due to displacement or blocking of Slug binding to the Epal due to occupancy by other Epal-binding repressor proteins. This is in part supported by some increase in transcription in being observed on mutation of the Epal in E6 cells. Alternatively, access to the promoter by both activating and repressing transcription factors may be reduced more globally by a mechanism such as histone methylation.

It has been reported that proteins from other viruses can also directly alter DNMT activity to silence genes such as E-cadherin. Latent membrane protein 1 (LMP1) of Epstein-Barr virus (EBV) induces expression of DNMT1, 3a and 3b, to increase DNMT activity and hypermethylation of E-cadherin [44]. Recently, it was also shown that following infection of germinal centre (GC) B cells, EBV down-regulates DNMT1 and DNMT3B expression, whilst upregulating DNMT3A. This is thought to contribute to viral persistence by promoting flexibility in latent viral promoter usage and by affecting the methylation status of several cellular genes with high CpG content [45]. The E1A oncoprotein of adenovirus type 5 binds directly to DNMT1 with a proposed conformational change to the enzyme, promoting the binding of DNA and S-adenosyl-L-methionine [46], [47] to increase methylation. Furthermore, evidence for induction of expression of DNMT1 by the HPV oncogenes E6 [29] and E7 [17], [46] adds to a body of supporting evidence for viral regulation, in particular for tumour viruses, of DNMT.

Others have reported that E7 suppresses E-cadherin transcription by increasing DNMT1 activity and inhibition of DNMT1 activity restores E-cadherin expression [16], [17]. Consistent with this we observed decreased E-cadherin in E7 expressing cells, which translated into a functional repression of E-cadherin mediated cellular aggregation. Furthermore, expression of E7 in E6 expressing cells resulted in a further drop in E-cadherin expression and E-cadherin mediated adhesion. This suggests that further activation of DNMTs may occur when both E6 and E7 are expressed, augmenting E-cadherin repression, or alternatively that either E6 or E7 may regulate E-cadherin through another pathway in addition to methylation dependent repression.

There is a body of evidence for regulation of E-cadherin by cancer-related viruses. Both hepatitis B-encoded X antigen and hepatitis C core antigen are associated with hypermethylation of the E-cadherin promoter and hepatocarcinogenesis [48], [49] and EBV reduces transcription of *CDH1* [50]. Viral repression of E-cadherin therefore may be important for viral carcinogenesis. For HPV, E-cadherin is reduced in tissues from patients infected with either low or high-risk HPV types [51], suggesting that its regulation is independent of the oncogenic potential of the virus.

HPV has a limited genetic repertoire of only eight ORFs but despite this, has a remarkable ability to redirect cellular pathways to its advantage. Cells infected with high-risk, cancer-causing HPV types are hyper-proliferative, anti-apoptotic and have reduced expression of markers required for immune detection. E-cadherin regulation by HPV is considered to contribute to viral immune evasion, as adhesion between keratinocytes and the epidermal antigen presenting cells, the LC, is E-cadherin-mediated [52]. HPV16 infected tissue has been reported to show reduced levels of surface E-cadherin [14], [53], which correlates with a reduction in LC density at the site of infection. The consequential reduction in viral antigen presentation is likely to be a contributing factor for HPV persistence in the immune competent host.

## Experimental Procedures

### Cell lines and reagents

HCT116 and HCT116 E6 cells [54] were cultured in Dulbecco's Modified Eagles Medium (DMEM) containing 2 mM L-glutamine (Sigma-Aldrich, MO, USA), penicillin, streptomycin and 10% heat inactivated fetal calf serum (FCS; PAA Laboratories, Austria) (cDMEM). Cells were incubated at 37 °C in a humidified incubator with 5% CO_2_. TSA (Sigma-Aldrich, MO, USA) was dissolved in ethanol and 5-Aza-dC (Merck, NZ) was dissolved in water. Unless otherwise stated, the following antibodies were used: mouse anti-human E-cadherin, Clone 36 (BD Biosciences) and HECD-1 (Takara Bio Inc., Japan); goat anti-actin (Santa Cruz, CA, USA); horseradish peroxidase (HRP) conjugated rabbit anti-mouse Ig (Dakocytomation, Denmark); HRP anti-goat IgG (Sigma).

### Confocal Microscopy

Cells were plated on poly-l-lysine (Sigma, CA, USA) treated glass coverslips and incubated overnight. Coverslips were washed, and cells fixed and permeabilised in −20 °C methanol for 4 min then incubated with an Alexa 488-labelled mouse anti-E-cadherin (clone 36) and 4′,6-diamidino-2-phenylindole (DAPI) for 1.5 h at RT. Cells were mounted in Slowfade Gold (Invitrogen) and imaged using a Zeiss LSM710 laser-scanning microscope.

### Tissue staining

Formalin-fixed paraffin-embedded tissues were de-waxed in xylene and rehydrated in graded alcohol. For antigen retrieval, sections were microwave-treated in Tris-HCl pH 9.5 containing 5% urea at 800 W for 2×10 min. Tissues were blocked in 10% fetal calf serum then incubated with alexa-488 labeled anti-E-cadherin antibody (clone 36; BD Biosciences) and DAPI. Tissues were mounted in Slowfade Gold and visualized using a BX51 Olympus fluorescent microscope.

### Homotypic adhesion assay

Cells were transfected with pEGFP constructs containing the E5, E6 or E7 genes from HPV16 and assayed for surface E-cadherin and adhesion as described previously [14]. Briefly, cells were trypsinised to remove surface E-cadherin and incubated in low adhesion plates for 2 h, then harvested and stained for E-cadherin using the mouse HECD-1 monoclonal antibody and allophycocyanin (APC-conjugated) donkey anti-mouse secondary antibody (Jackson Immuno Research Laboratories Inc., PA, USA). Cells were gated for equivalent levels of green fluorescent protein (GFP) expression and 2000 events were acquired. Surface E-cadherin expression on the GFP positive events was measured by flow cytometry and the level of E-cadherin mediated aggregation was assessed using forward scatter.

### Luciferase reporter assay

The pGL3 plasmid with the entire human E-cadherin regulatory region and a series of deletion constructs [28], all with the *Firefly* luciferase reporter, were used in this study. Cells at 8×10^5^ cells/well in six-well plates were transiently transfected with a total of 2 µg DNA containing 1 µg of the test plasmid, 10 ng of the pRL-SV40 vector expressing *Renilla* luciferase and 990 ng of irrelevant filler DNA (pCDNA3) in 6 µl of polyethylenimine (Polysciences, PA, USA). Cells were harvested 48 h later, and extracts were assayed using the Firefly and Renilla Luciferase Assay Kit (Biotium, Inc, CA, USA) and all data was adjusted for transfection efficiency based on the Renilla signal.

### mRNA quantification by real-time reverse transcription PCR

RNA was extracted from cells using the RNeasy mini kit (Qiagen, CA, USA), and reverse transcribed into cDNA using SuperScript polymerase (Invitrogen, CA, USA). SYBR Green reverse transcription (RT) quantitative PCR (qRT-PCR) was carried out using the following primers: E-cadherin, GAAGGTGACAGAGCCTCTGGAT and GATCGGTTACCGTGATCAAAATC and glyceraldehyde-3-phosphate dehydrogenase (GAPDH), CCCACTCCTCCACCTTTGAC and TTGCTGTAGCCAAATTCGTTGT. qRT-PCR was performed using the Applied Biosystems 7500 Real-Time System. The ΔΔC_T_ method was used to calculate fold change in mRNA between samples for genes of interest, with GAPDH as the reference gene.

### Western blotting

Cells were harvested in lysis buffer (40 mM Tris pH 7.4, 150 mM NaCl, 0.01% Triton X100, 1 mM EDTA) containing protease inhibitor cocktail (Roche). Proteins were resolved by SDS-PAGE and electroblotted onto Hybond C+ nitrocellulose membrane (GE Healthcare, NJ, USA). Membrane was blocked overnight at 4 °C in 5% skim milk and incubated with primary antibodies for 1.5 h at RT followed by HRP conjugated secondary antibodies for 1 h. Chemiluminescence signal was detected following incubation with SuperSignal® West Pico luminal/enhancer solution (Perbio, IL, USA).

### Bisulfite conversion of genomic DNA

Bisulfite sequencing was performed as previously described [17]. Briefly, genomic DNA was denatured and cytosines were sulfonated using an EZ DNA Methylation-Gold Kit (ZymoResearch, CA, USA). The modified DNA was subjected to PCR amplification of the 25 CpG-containing minimal E-cadherin promoter sequence. PCR products were ligated into the pGEM-T Easy vector and following transformation ten colonies were selected for sequencing.

### DNMT activity assay

Cells were seeded at 4×10^6^ cells in 75 cm^2^ flasks. The following day cells were harvested, counted and nuclear extracts prepared using EpiQuick™ Nuclear Extraction Kit I (Epigentek, NY, USA). Nuclear extracts were quantified using the Bradford BCA Protein Quantification Kit (Pierce, Thermo Fischer Scientific, Rockford, IL, USA). One µL of nuclear extract (containing between 5–20 µg of protein) was then assayed for nuclear activity using EpiQuick™ DNA Methyltransferase Activity/Inhibitor Screening Assay Kit (Epigentek).

### Chromatin immunoprecipitation

Chromatin immunoprecipitation (ChIP) was performed using a protocol from Millipore, with minor modifications. Briefly, crosslinking was undertaken by treating cells with 1% formaldehyde for 10 min at 37 °C, followed by quenching of the reaction using 125 mM glycine for 5 min at RT. Cells were harvested and resuspended in 550 µl SDS lysis buffer (1% SDS, 10 mM EDTA, 50 mM Tris-HCl pH 8.1 and Sigmafast protease inhibitor cocktail (Sigma Aldrich)), and incubated on ice for 10 min. Lysates were sonicated to yield DNA fragments of sizes between 0.5 and 1 kb, and the debris removed by centrifugation. 200 µL of the sample was used as ‘input’, while dilution buffer (0.01% SDS, 1.1% Triton X-100, 1.2 mM EDTA, 16.7 mM Tris-HCl, pH 8.1, 167 mM NaCl, and protease inhibitors) was added to the remaining sample to a final volume of 1 mL. Samples were precleared with Sepharose A/G beads (Invitrogen) that had been blocked with 1 mg/mL salmon sperm DNA and 1 mg/mL BSA. Immunoprecipitation was undertaken overnight at 4 °C with 4 µg of antibody against AML1 (N-20, Santa Cruz Biotechnology), Slug (H-140, Santa Cruz Biotechnology), Sp1 (1C6, Santa Cruz Biotechnology) or negative control EE epitope tag antibody, in the presence of blocked Sepharose A/G beads. After a series of washes, complexes were eluted from the beads with 1% SDS and 0.1 M NaHCO_3_ for 30 min at RT, and crosslinking was reversed by 0.2 M NaCl at 65 °C for 4 h. Proteins were digested with 20 µg proteinase K (Invitrogen) for 1 h at 45 °C, and the DNA recovered using a PCR purification kit (Invitrogen). PCR was undertaken using primers against the E-cadherin regulatory region: forward 5′- CTGATCCCAGGTCTTAGTGAG -3′ and reverse 5′- GGGCTGGAGTCTGAACTGAC -3′.

### Statistical analysis

Data are presented as mean ± standard error of the mean. n = number of independent experimental replicates. One-way analysis of variance (ANOVA) with Tukey's post-test was used for comparison between three or more groups. Paired t-test was used for comparison between two groups.
